# Updates in Skin Cancer in Transplant Recipients and Immunosuppressed Patients: Review of the 2022–2023 Scientific Symposium of the International Immunosuppression and Transplant Skin Cancer Collaborative

**DOI:** 10.3389/ti.2024.12387

**Published:** 2024-03-18

**Authors:** Catherine Pisano, Justin J. Leitenberger, Melissa Pugliano-Mauro, Bryan T. Carroll

**Affiliations:** ^1^ Department of Dermatology, Brigham and Women’s Hospital, Boston, MA, United States; ^2^ Department of Dermatology, Oregon Health & Science University, Portland, OR, United States; ^3^ Department of Dermatology, University of Pittsburgh Medical Center, Pittsburgh, PA, United States; ^4^ Department of Dermatology, Case Western Reserve University School of Medicine, Cleveland, OH, United States

**Keywords:** solid organ transplant (SOT), immunosuppression, chemoprophylaxis, skin cancer, checkpoint inhibitors

## Abstract

The International Immunosuppression and Transplant Skin Cancer Collaborative (ITSCC) and its European counterpart, Skin Care in Organ Transplant Patients-Europe (SCOPE) are comprised of physicians, surgeons, and scientist who perform integrative collaborative research focused on cutaneous malignancies that arise in solid organ transplant recipients (SOTR) and patients with other forms of long-term immunosuppression. In October 2022, ITSCC held its biennial 4-day scientific symposium in Essex, Massachusetts. This meeting was attended by members of both ITSCC and SCOPE and consisted of specialists including Mohs micrographic and dermatologic oncology surgeons, medical dermatologists, transplant dermatologists, transplant surgeons, and transplant physicians. During this symposium scientific workshop groups focusing on consensus standards for case reporting of retrospective series for invasive squamous cell carcinoma (SCC), defining immunosuppressed patient status for cohort reporting, development of multi-institutional registry for reporting rare tumors, and development of a KERACON clinical trial of interventions after a SOTRs’ first cutaneous SCC were developed. The majority of the symposium focused on presentation of the most up to date research in cutaneous malignancy in SOTR and immunosuppressed patients with specific focus on chemoprevention, immunosuppression regimens, immunotherapy in SOTRs, spatial transcriptomics, and the development of cutaneous tumor registries. Here, we present a summary of the most impactful scientific updates presented at the 2022 ITSCC symposium.

## Introduction

The International Immunosuppression and Transplant Skin Cancer Collaborative (ITSCC) was established by dermatologic surgeons in 2000, and its European counterpart, Skin Care in Organ Transplant Patients-Europe (SCOPE), was established by dermatologists and transplant physicians in 2001. Both ITSCC and SCOPE are now comprised of physicians, surgeons, and scientist who perform integrative collaborative research focused on cutaneous malignancies that arise in solid organ transplant recipients (SOTR) and patients with other forms of long-term immunosuppression. In October 2022, ITSCC held its biennial 4-day scientific symposium in Essex, Massachusetts. This meeting was attended by members of both ITSCC and SCOPE and consisted of 36 specialists including Mohs micrographic and dermatologic oncology surgeons, medical dermatologists, transplant dermatologists, transplant surgeons, transplant physicians, fellows-in- training, and residents-in-training. The symposium attendees were from all over the United States as well as several different European countries, and included physicians from large academic institutions as well as private practices. During this symposium a multi-disciplinary tumor-board for complex clinical cases was held, a keynotes lecture by Dr. Matthew Bottomley from the University of Oxford on the development of a secondary malignancy in SOTRs with cutaneous SCC was given, and scientific workshop groups focusing on consensus standards for case reporting of retrospective series for invasive squamous cell carcinoma (SCC), defining immunosuppressed patient status for cohort reporting, development of multi-institutional registry for reporting rare tumors, and development of a KERACON clinical trial of interventions after a SOTRs’ first cutaneous SCC were developed. The majority of the symposium focused on presentation of the most up to date research in cutaneous malignancy in SOTR and immunosuppressed patients with specific focus on chemoprevention, immunosuppression regimens, immunotherapy in SOTRs, spatial transcriptomics, and the development of cutaneous tumor registries. Here, we present a summary of the most impactful scientific updates presented at the 2022 ITSCC symposium.

## Chemoprevention

Chemoprevention, defined as the use of prophylactic medical management to prevent the development of malignancies, specifically in the context of cutaneous SCC in SOTRs was a largely discussed topic during the ITSCC 2022 symposium. While it is well-known that SOTRs have a 20 to 200 times higher risk of developing cutaneous SCC and increased mortality compared to non-SOTR patients, primary prevention of cutaneous SCC is quickly becoming one of the most important roles of dermatologic care in the overall health of SOTR [1]. However, no consensus guidelines on chemoprevention of cutaneous SCC in SOTRs previously existed, until the 2021 publication of the Delphi Consensus Statement by Massey et al. [2].

At the biennial ITSCC symposium in September 2018, several experts in transplant dermatology began the process of developing a Delphi study to provide consensus-based recommendations for the prevention of cutaneous SCC in SOTRs, of which the results were published in JAMA Dermatology by Massey et al. in 2021. The panel of experts involved in this Delphi study represented 13 countries with 56% of those panelists located in the United States. Additionally, this Delphi study used a threshold of 80% or higher to define consensus. The results of this study showed that there is consensus recommendation for routine skin cancer surveillance exams in all SOTRs [2]. A 2019 Delphi consensus recommended all high-risk Caucasian SOTRs should be screened within 2 years of the solid organ transplant whereas all non-high-risk Caucasians, Asian, Hispanic, and high-risk African American patients should be screened within 5 years of solid organ transplantation. High-risk transplant patients were defined in this Delphi as thoracic organ transplant recipients, age 50 or above at time of solid organ transplantation, and male SOTRs [3]. Additionally, in keeping with previously published studies, regular use of sunscreen and sun-protective behaviors was recommended by this expert panel [2–4].

In regards to topical treatment of precancerous lesions, specifically actinic keratoses, the Delphi study resulted in full consensus for lesion-directed therapy using cryotherapy for scattered actinic keratoses and the use of field therapy (with or without the adjuvant use of cryotherapy for thicker lesions) for actinic keratoses confined to a single anatomic location. While no full-consensus was reached in regards to which topical agent should be used for field therapy in this setting, this study had a near-consensus (70% to less than 80% agreement) in favor of using topical fluorouracil. Additionally, while 74% of the group reported that photodynamic therapy (PDT) had the best adherence, only 4% considered PDT to be the most effective field agent.

In regards to oral chemoprevention, the only agent that had a consensus recommendation in this study was acitretin in the setting of high-rate of development of cutaneous SCC (>10 tumors per year) or development of high-risk cutaneous SCC (AJCC8 T3 or above or Brigham and Women’s Hospital stage T2b or above) in SOTR, which is supported by prior findings in randomized controlled trials involving renal transplant recipients [5, 6]. However, no consensus was reached in regards to chemoprevention after the development of a first low-risk SCC in SOTR, regardless of specific organ transplanted (i.e., abdominal vs. thoracic). Additionally, no consensus recommendation for use of oral nicotinamide or capecitabine was reached.

This study also had a consensus recommendation to discuss change in SOTRs’ immunosuppression regimen with the transplant team in the setting of advanced cutaneous SCC, defined in this study as multiple invasive low risk cutaneous SCCs (>10 tumors per year) or development of a high-risk cutaneous SCC. No consensus recommendation was made in regards to which specific immunosuppression regimen should be used to provide the lowest risk for development of cutaneous SCC.

The findings of the Massey et al. Delphi study detailing consensus-based recommendations for the prevention of cutaneous SCC in SOTR were heavily discussed during the ITSCC 2022 symposium, and provide the most up-to-date findings supporting preventative dermatologic care in this patient population. Additional presentations at this symposium discussed ongoing research into the use of acitretin, capecitabine, and nicotinamide for chemoprevention in SOTR, emphasizing the importance of continued work in this area to find the most effective prevention for cutaneous SCC in transplant recipients given the increased risk of morbidity and mortality in this patient population.

## Immunosuppression

Chronic immunosuppression is an important part of long-term medical treatment of SOTRs, and the increased risk of developing cutaneous malignancies in this setting is well documented and an area in which ongoing research is aiming to minimize. The role of immunosuppression, development of cutaneous malignancies, and possible alterations of immunosuppression therapy was a heavily discussed topic at the ITSCC 2022 symposium.

A recent publication polled expert transplant dermatologist to determine which immunosuppression regimen was clinically correlated with the development of the most cutaneous malignancies, and 88% of respondents reported azathioprine was associated with development of the most cutaneous malignancies following solid organ transplantation. Additionally, 69% reported that sirolimus was the immunosuppressant least associated with the development of cutaneous malignancies following transplant [2]. These clinical findings are supported by transitional research showing that cutaneous SCC in SOTRs taking azathioprine show unique mutational signatures caused by UVA absorption by DNA [7, 8]. While azathioprine is now less commonly used as a primary immunosuppression regimen for SOTRs, it is still used in patients who are intolerant to mycophenolate therapy or who may be planning to become pregnant [9].

More modern immunosuppression regimens appear to have a decreased risk of developing cutaneous malignancies when compared to azathioprine. A recent large retrospective control-matched cohort study by Gibson et al. published in 2021 found a significant reduction in skin cancer development with the use of tacrolimus and mycophenolate when compared to cyclosporine and azathioprine, respectively [10]. This study found an incidence rate ratio (IRR) of 0.44 (*p* = 0.03, 95% CI = 0.21–0.92) when SOTRs were switched from cyclosporine to tacrolimus, as well as an IRR of 1.66 (*p* = 0.01, 95% CI = 1.16–2.36%) with azathioprine compared to an IRR of 0.78 with mycophenolate (*p* = 0.18, 95% CI = 0.54–1.12) [9]. Additionally, transition from azathioprine to mycophenolate appears to reduce the risk of developing a first cutaneous SCC post solid organ transplantation with mycophenolate having an IRR of 0.49 (*p* = 0, 95% CI = 0.32–0.75) compared to azathioprine in this setting [9–11].

Another point of discussion regarding immunosuppressive regimens was the more recent use of belatacept as a primary immunosuppressant or adjuvant immunosuppressant used with calcineurin inhibitors in kidney transplant recipients and the correlation to the development of skin cancer. Given the more recent incorporation of belatacept in SOTR medical management, there is currently limited evidence, but thus far, small single-center studies show that use of belatacept in the place of calcineurin inhibitors leads to a lower risk of developing skin cancers post solid organ transplantation [12].

As discussed above in the Massey et al. publication, the development of multiple invasive low risk cutaneous SCC or the development of a high-risk cutaneous SCC should prompt discussion of alteration of immunosuppression regimen with the patient’s transplant team. The two secondary prevention strategies used in this situation are to change the immunosuppressive regimen or change the immunosuppressive intensity, and individual patient assessment must be used to determine the best course of action [9]. As immunosuppression regimens continue to evolve, more research is needed to determine best therapy and dose to balance maintaining the function of the transplanted organ and decreasing the risk of secondary malignancies.

## Checkpoint Inhibitor Therapy in SOTR

Given the increased risk of SOTRs to development high-risk cutaneous malignancies that may require systemic treatment, the use of immune checkpoint inhibitors (ICIs) in this population is a highly discussed topic at this time. The use of ICIs in SOTR with high-risk cutaneous malignancies, including melanoma, Merkel cell carcinoma, and high-risk SCC, presents a significant challenge as ICIs place transplant recipients at risk of acute allograft rejection. Historically, SOTRs have been excluded from ICI clinical trials for treatment of advanced skin cancers as retrospective studies have shown acute allograft rejection rates between 10% and 65% with ICI use in this population [13]. Of these SOTRs who experience acute allograft rejection with ICI therapy, 24%–81% subsequently lose their allograft, which may lead to death [13, 14].

Careful deliberation and risk-benefit assessment is needed on an individualized basis when considering ICI treatment in SOTRs. While ICIs are the only approved systemic treatments for locally advanced or metastatic cutaneous SCC and Merkel cell carcinoma, the risk of acute leading to fulminant rejection of the allograft is a major factor in oncologic management of SOTR [15, 16]. Given the high risk of allograft rejection, kidney transplant recipients are the primary SOTRs that may be considered for ICI treatment in the setting of advanced cutaneous malignancy as transplant rejection can be managed by dialysis in most cases and rarely leads to fatality. However, thoracic transplant patients (i.e., heart or lung) are less commonly considered for ICI therapy in this setting as risk of allograft failure is more life-threatening [13]. While retrospective and systematic reviews are helpful in assessing the risk of ICI use in SOTR, there is now a major focus on prospective and randomized controlled trials in this area to better elucidate the role of ICI in SOTRs. The ongoing CASE (Cemiplimab-rwlc Survivorship and Epidmiology) study is a longitudinal prospective multicenter study evaluating the safety and effectiveness of cemiplimab used to treat advanced cutaneous SCC in SOTRs. Preliminary results from the CASE study appear to be similar to those from prior ICI trials that excluded SOTRs [15, 16]. Additionally, an active phase I trial studying the efficacy and risk of tacrolimus, nivolumab, and ipilimumab in treating kidney transplant recipients with selected unresectable or metastatic cancers (including cutaneous SCC) was discussed at the ITSCC 2022, and importance of such trials was emphasized [17, 18].

The use of ICI in advanced cutaneous malignancies is a mainstay of therapy in treating non-immunocompromised patients, and is now playing a more prominent role in the treatment of such malignancies in SOTR. More prospective and randomized controlled studies are needed to elucidate the role of ICI in SOTR, specifically in regards to which SOTRs are good candidates for ICI therapy, patient factors that may help predict allograft rejection, immunosuppressive regimens that may be protective of the allograft during ICI treatment, and ideal ICI dosing to provide oncologic benefit while reducing risk of allograft rejection.

## Spatial Transcriptomics

Spatial transcriptomics, particularly in regards to cutaneous malignancies, was another highly discussed topic at the 2022 ITSCC symposium given the increase in popularity of spatial transcriptomics in cancer research as of late. In brief, spatial transcriptomics allows for the measure of gene activity in a tissue sample while allowing mapping of where the activity is occurring without disrupting the anatomic structure of the sampled tissue [19, 20]. Previously, bulk and single-cell RNA sequencing were used to better understand cell to cell interactions in cancer; however, these techniques did not allow for retention of spatial orientation in the tissue specimens [19]. Given the importance of the tumor microenvironment, tumor heterogenicity, and tumor interface in cancer, spatial transcriptomics have provided significant advances in the microscopic understanding of malignancies and is an exciting advancement in oncologic research. With better understanding of spatial histology of tumors, there is greater potential to improve pathologic diagnosis, understanding of prognostic factors, understanding of tumorigenesis and progression, and prediction of treatment response [19, 20].

Spatial transcriptomics is now being used in the research of high-risk and advanced cutaneous malignancies, including those seen in SOTRs. Specifically, Dr. Matthew Bottomley of the University of Oxford is using spatial transcriptomic profiling to explore immunosuppression and immunosenescence-driven skewing of immune response in cutaneous SCCs in kidney transplant recipients, which may explain the enhanced predisposition to cutaneous SCC in these cohorts [21]. Additionally, Dr. John Carucci of New York University presented his lab’s research on the transcriptomic profile of CDS+ tumor infiltrating lymphocytes (TIL) in aggressive basal cell carcinomas, and the subsequent effects on T-cell trafficking, clonal expansion, and T-cell exhaustion. This work is an exciting advancement in the further understanding of high-risk cutaneous malignancies on a microscopic and genetic level, and encourages further research in the area of high-risk cutaneous malignancies using spatial transcriptomics.

## Tumor Registries

Tumor registries specific to cutaneous malignancies was also a prominent topic at the 2022 ITSCC symposium. While melanoma is often included in national tumor registries such as the National Cancer Institute’s Surveillance, Epidemiology, and End Result Program, other primary cutaneous malignancies are not. This lack of tumor registry, specifically for rare cutaneous tumors, inhibits the further understanding of incidence, prognosis, natural history of disease, and treatment response. While several academic institutions in the United States and Europe have individual tumor registries, there has yet to be a national or international registry to collect combined information on these tumors. One of the focus groups at the ITSCC 2022 symposium was dedicated to initiating a multi-institution registry for rare cutaneous tumors. This registry plans to collect information about the tumors as well as patient characteristics including history of solid organ transplants and immunosuppression status. Not only will a national, and perhaps 1 day international, tumor registry of rare cutaneous malignancies provide a larger sample size of such tumors to allow a better understanding of innate characteristics of these cancers, but it will also elucidate the relationship between SOTR, immunosuppression, and the development and outcome of these rare tumors.

## Future Direction

The ITSCC 2022 symposium was a great success, and provided an opportunity for experts in the field of transplant and immunosuppression dermatology to discuss the most recent scientific advancements in this area and collaborate on ongoing research. Through the workgroups developed during this meeting, longitudinal projects including development of a multi-institutional registry for reporting rare tumors, consensus definition of immunosuppressed patient status for cohort reporting, consensus standards for case reporting of retrospective series for invasive SCC, and KERACON clinical trial of interventions after a SOTR first cutaneous SCC are currently underway. Additionally, ITSCC is now offering a 2-year academic mentorship program which connects junior members of ITSCC with more established senior members of ITSCC to assist in career and academic development, particularly in regards to establishing transplant and immunosuppressed patient clinics as well as performing clinical and/or translation research in these areas. Further discussion and presentation of scientific work in the dermatologic care in SOTRs and the immunosuppressed patient population are scheduled for the annual ITSCC meeting at the American Academy of Dermatology annual meeting as well as the SCOPE symposium planned for Fall 2023, and the next ITSCC biennial symposium in Fall 2024.
